# Impact of influenza vaccine on childhood otitis media in Taiwan: A population-based study

**DOI:** 10.1371/journal.pone.0190507

**Published:** 2018-01-05

**Authors:** Pei-Wen Wu, Chien-Chia Huang, Wei-Chieh Chao, Chi-Chin Sun, Cheng-Hsun Chiu, Ta-Jen Lee

**Affiliations:** 1 Department of Otolaryngology–Head and Neck Surgery, Chang Gung Memorial Hospital and Chang Gung University, Keelung, Taiwan; 2 Division of Rhinology, Department of Otolaryngology, Chang Gung Memorial Hospital and Chang Gung University, Taoyuan, Taiwan; 3 Graduate Institute of Clinical Medical Sciences, College of Medicine, Chang Gung University, Taoyuan, Taiwan; 4 Department of Ophthalmology, Chang Gung Memorial Hospital and Chang Gung University, Keelung, Taiwan; 5 Department of Pediatrics, Chang Gung Memorial Hospital and Chang Gung University, Taoyuan, Taiwan; 6 Molecular Infectious Disease Research Center, Chang Gung Memorial Hospital and Chang Gung University, Taoyuan, Taiwan; Harvard School of Public Health, UNITED STATES

## Abstract

**Purpose:**

Acute otitis media (AOM) is a common infectious disease in children and usually accompanied by a preceding viral respiratory tract infection, especially in the preschool-age population. The study aimed to evaluate impact of influenza vaccine on childhood otitis media.

**Methods:**

This retrospective cohort study included data for 803,592 children (<10 years old) recorded in Taiwan’s National Health Insurance Research Database. AOM incidence and tympanostomy tube insertion incidence in each influenza season before and after the introduction of traditional injectable trivalent influenza vaccine (TIV) were compared using the Poisson regression analysis to estimate the incidence rate ratios (IRRs) and 95% confidence intervals (CIs).

**Results:**

In children < 2 years old, the age group eligible for free influenza vaccination, there was a significant reduction in seasonal AOM incidence after TIV introduction in 2004 (from 98.4 episodes/1000 person-seasons [95% CI: 96.4–100.5] to 66.1 episodes/1000 person-seasons [95% CI: 64–68.1]). In addition, with the increased vaccine coverage rate, the outpatient visits for AOM in the influenza season of 2005 and 2006 were significantly lower than that in 2004 (IRR = 0.85 and 0.80, respectively, p < 0.0001).

**Conclusions:**

A significant reduction in primary care consultations for children <2 years old was observed after the introduction of the TIV in Taiwan in 2004. With the increased vaccine coverage, there was an additional decline in 2005 and 2006. In addition of the direct protection provided by the vaccination, we believe that TIV may have induced some herd immunity that further contributed to the reduction in influenza attack rates and the rates of associated AOM in that age group. These reductions were observed only in vaccine-eligible children, while older children, who were not enrolled in the influenza vaccination program during the study period, have experienced increases in the AOM incidence during the 2004–2006 period compared to the 2000–2003 period.

## Introduction

Acute otitis media (AOM) is a common infectious disease among children, especially in the preschool-age population. The majority of studies regarding childhood AOM reported that the incidence peaked in children 6–12 months old, and decreased with age [1–8]. AOM is also among the leading causes for children’s visits to physicians and is the most common reason for prescription of antibiotics to children with respiratory conditions [9]. Moreover, otitis media with effusion (OME) can occur as a post-inflammatory response to AOM. The effusion in the middle ear could decrease sound transmission, resulting in conductive hearing loss [10]. Tympanostomy tube insertions are recommended for children with bilateral chronic OME (OME lasting for 3 months or longer) and recurrent AOM with middle ear effusion [11]. The resulting psychological burden on parents and substantial economic costs also demonstrate that AOM is a major public health concern [12].

There is evidence that viruses play a crucial role in the development of AOM [13–15]. AOM is usually accompanied with a preceding viral upper respiratory tract infection (URI), which may initiate a cascade of events that eventually leads to inflammation of the middle ear mucosa [13]. AOM may be regarded as a complication of this viral syndrome. The impact of influenza vaccination on childhood otitis media has been discussed worldwide, but no consensus has been reached to date.

Since 2004, the Taiwan Center for Disease Control (CDC) launched a program to provide free influenza vaccine (traditional injectable trivalent influenza vaccine, TIV) for children aged 6–24 months. Additionally, pneumococcal conjugate vaccines (PCVs) had not become popular in Taiwan until 2007.

In this study, we used the Taiwan National Health Insurance Research Database (NHIRD) to assess trends in the incidence of childhood AOM and tympanostomy tube insertion during the influenza season. The NHIRD contains claims data derived from the government-operated, single-payer National Health Insurance program, which was launched in 1995 and covers nearly all (99%) of the 23 million residents of Taiwan [16]. Every admission, operation, and outpatient visit record is included in this database. Our study aim was to assess the efficacy of influenza vaccine in preventing AOM over a 7-year period that encompassed the introduction of TIV in Taiwan, since October 2004.

## Materials and methods

We conducted a retrospective cohort study to assess secular trends in the incidence of childhood AOM and tympanostomy tube insertion in each influenza season before and after the introduction of the TIV in Taiwan, by using the NHIRD. The influenza season is considered to run from October through March, as suggested by the Taiwan CDC [17]. The tympanostomy tube insertion procedure is considered a surrogate of refractory and recurrent AOM with OME. The data underlying this study is from the National Health Insurance Research Database (NHIRD), which has been transferred to the Health and Welfare Data Science Center (HWDC). Interested researchers can obtain the data through formal application to the HWDC, Department of Statistics, Ministry of Health and Welfare, Taiwan (http://dep.mohw.gov.tw/DOS/np-2497-113.html). The NHIRD includes no personal information, and this study was reviewed and approved by the Ethics Institutional Review Board of Chang Gung Memorial Hospital.

We included the NHIRD data of all children younger than 10 years. We stratified these children into three age groups: < 2 years old, 2–4 years old, and 5–9 years old. We used the International Classification of Diseases, Ninth Revision, Clinical Modification (ICD-9-CM) to distinguish AOM-related medical utilization. We identified children with AOM episodes by searching for the relevant ICD-9-CM codes (3810, 38100, 38101, 38102, 38103, 3820, 38200, 38201, 38202) in each outpatient visit. We identified children with tympanostomy tube insertion by searching for the procedure codes for myringotomy with ventilation tube insertion under a microscope (84015B). To avoid confounding by PCV, which was not popular in Taiwan before 2007 [18], our study period included only seven influenza seasons, between October 2000 and March 2007.

The incidence of AOM was defined as the total number of outpatient visits during the influenza season, divided by the total person-season of the study population during the time period. The incidence of tympanostomy tube insertion was defined as the total number of operative procedures performed during the influenza season, divided by the total person-season of the study population during the study period. A 3-month screening period was set from the first operative procedure and any subsequent operation during the screening period was considered as a revision operation for the same episode. Age-specific incidence was calculated for the following age groups: < 2 years old, 2–4 years old, and 5–9 years old. The 95% confidence intervals (CIs) and incidence rate ratios (IRRs) were estimated using Poisson regression models. Statistical analyses were performed using SPSS Version 18.0 (SPSS Inc., Chicago, IL, USA).

## Results

The study population comprised 803,592 children < 10 years old in influenza seasons between October 2000 and March 2007. There were 117,591 outpatient visits with AOM identified in 49,038 children. During the pre-TIV era (2000–2003), the mean seasonal AOM incidence was 58.9 episodes/1000 person-seasons (95% CI: 55.6–62.1) in those <10 years old, with the highest incidence in those < 2 years old (98.4 episodes/1000 person-seasons; 95% CI: 96.4–100.5), followed by those 2–4 years old (61.7 episodes/1000 person-seasons; 95% CI: 60.7–92.6), and those 5–9 years old (28.6 episodes/1000 person-seasons; 95% CI: 28.0–29.1). After TIV introduction (2004–2006), the mean seasonal AOM incidence was 67 episodes/1000 person-seasons (95% CI: 63.4–70.6) in those < 10 years old, with the highest incidence in those 2–4 years olds (71.7 episodes/1000 person-seasons; 95% CI: 70.2–73.1), followed by those < 2 years old (66.1 episodes/1000 person-seasons; 95% CI: 64.0–68.1), and those 5–9 years old (33.8 episodes/1000 person-seasons; 95% CI: 33.1–34.4). The incidence of AOM in childen <2 years old declined by 32.8% after introduction of TIV. Additionally, the incidence of AOM in those 2–4 years old and 5–9 years old were both increased by 16.2% and 18.2%, respectively.

**Fig 1:**
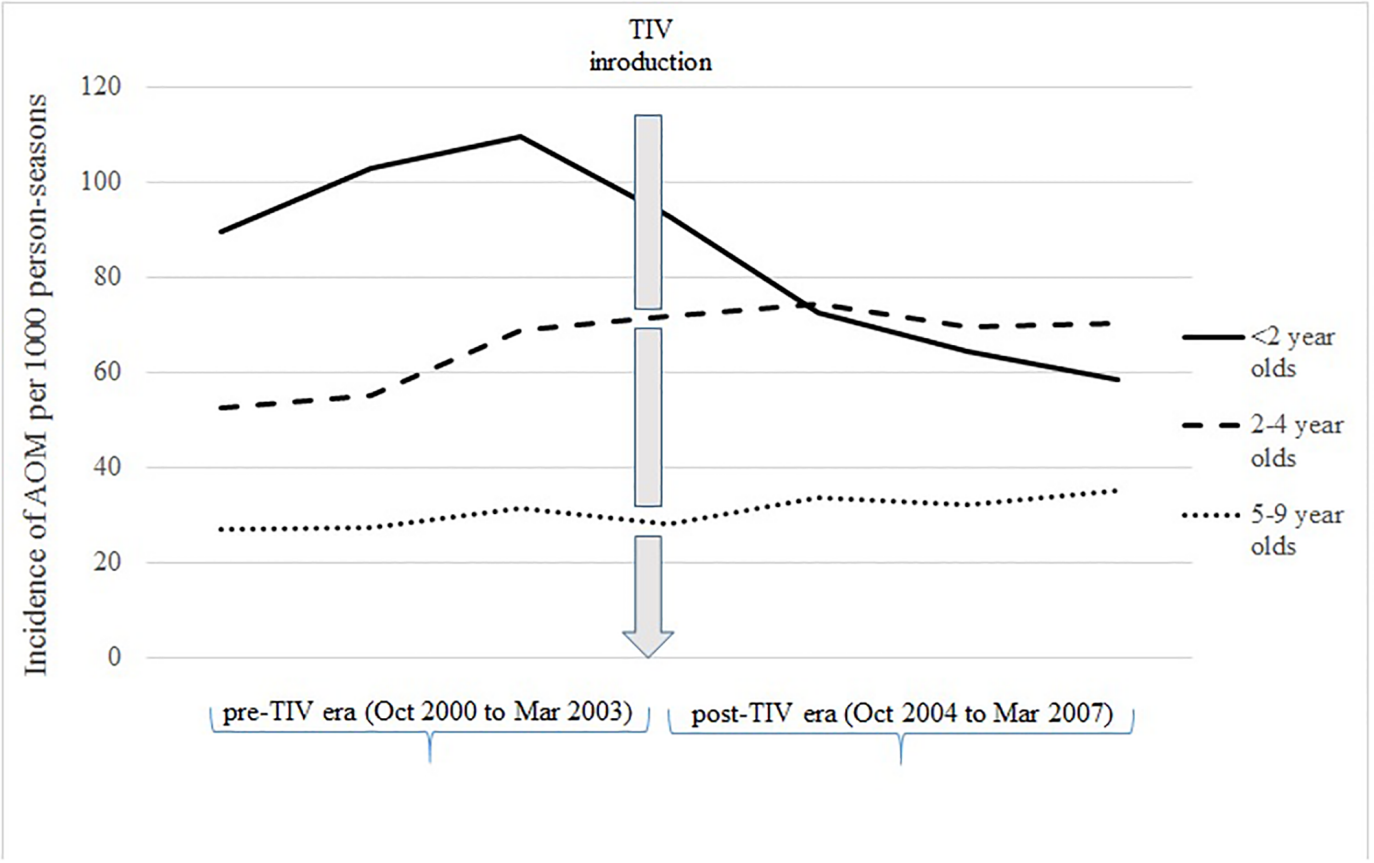
Significant reduction in AOM incidence was observed after introduction of the trivalent influenza vaccine (TIV). In children < 2 years old, who received free influenza vaccination, there was a significant reduction in AOM incidence after the introduction of the TIV in 2004. Meanwhile, the seasonal incidence of AOM in those 2–4 years old and 5–9 years old in 2004–2006 were significantly increased.

In children < 2 years old, the age group that received free influenza vaccination, there was a significant reduction in AOM incidence after the introduction of the TIV in 2004 (Fig 1, Table 1). The number of outpatient visits for AOM in the influenza seasons of 2004–2006 were significantly lower than that in the seasons 2000–2003 (IRR = 0.71, 0.61, 0.57, in 2004, 2005, 2006, respectively, p < 0.0001). Moreover, compared to the pre-TIV era (2000–2003), the seasonal incidence of AOM in children 2–4 years old and 5–9 years old in 2004–2006 were significantly increased (Fig 1, Table 1).

**Table 1:** Incidence rate ratio (IRR) of outpatient visits with acute otitis media (AOM) in each age group in each influenza season after introduction of the traditional injectable trivalent influenza vaccine (TIV) in 2004.

	2000–2003	2004	2005	2006
< 2 years old	1.00	0.71*(0.69–0.74)	0.61*(0.58–0.63)	0.57*(0.54–0.59)
2–4 years old	1.00	1.19*(1.16–1.22)	1.10*(1.08–1.13)	1.09*(1.06–1.11)
5–9 years old	1.00	1.26*(1.23–1.33)	1.36*(1.33–1.40)	1.58*(1.53–1.62)

*p < 0.0001, (95% confidence interval)

**Fig 2:**
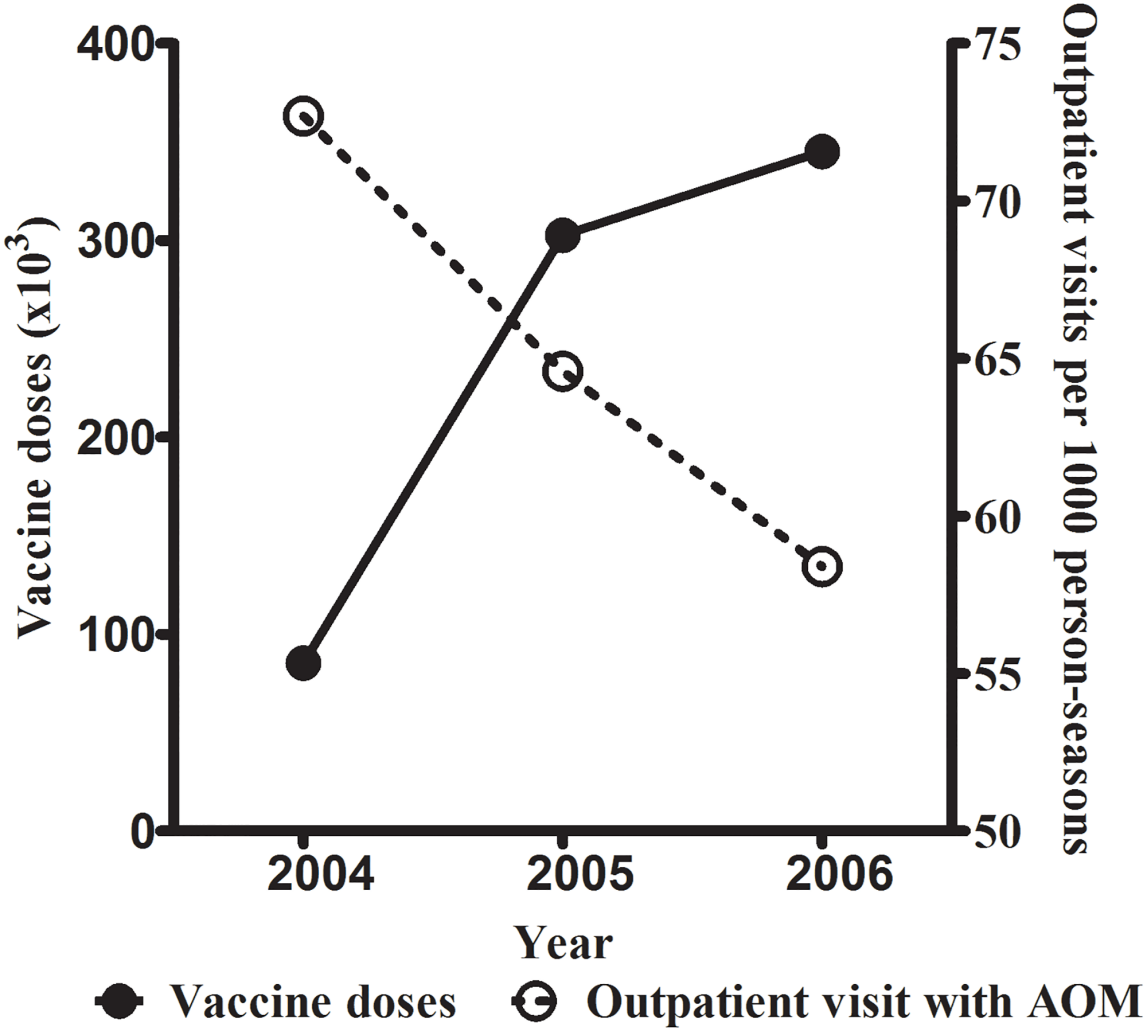
With the increased vaccine coverage rate, the incidence of AOM declined correspondingly. With the increased vaccine coverage, the number of outpatient visits for AOM in children < 2 years old in the influenza season in 2005 and 2006 was significantly lower than that in 2004 (IRR = 0.85 and 0.80, respectively, *P* < 0.0001).

In addition, with the increased vaccine coverage (Table 2) [19], the number of outpatient visits for AOM in children < 2 years old in the influenza season in 2005 and 2006 was significantly lower than that in 2004 (IRR = 0.85 and 0.80, respectively, p < 0.0001; Fig 2). Furthermore, we identified 678 tympanostomy tube insertions in 538 children. The IRR for each influenza season between October 2000 and March 2007 was not significantly different in all three age groups (Table 3).

**Table 2:** Influenza vaccine coverage rate in each influenza season.

	Fully vaccinated^a^ (%)	Partially vaccinated^b^ (%)	Non-vaccinated^c^ (%)
2004	38011 (25)	8747 (6)	106169 (69)
2005	149158 (42)	37289 (11)	166762 (47)
2006	197909 (36)	33273 (6)	314740 (58)

Data were adapted from Centers for Disease Control and Prevention, Taiwan.[19]

^*a*^ Patients who received two doses of vaccine in the primary year and one dose each subsequent year, or received at least one dose before and one dose in the particular season, were classified as fully-vaccinated.

^*b*^ Patients who received one dose of vaccine, but had never received a dose before were classified as partially vaccinated.

^*c*^ Patients who did not receive any dose of vaccine were classified as non-vaccinated.

**Table 3:** Incidence rate ratio (IRR) of tympanostomy tube insertion in each age group in each influenza season.

	2000	2001	2002	2003	2004	2005	2006
< 2 years old	1.00	1.63*(0.88–3.03)	0.68*(0.32–1.44)	0.96*(0.46–2.01)	1.20*(0.60–2.40)	1.59* (0.82–3.09)	1.59*(0.71–3.58)
2–4 years old	1.00	0.95*(0.58–1.55)	0.85*(0.50–1.15)	1.34*(0.84–2.13)	1.60*(1.01–2.52)	1.47*(0.91–2.37)	1.69*(1.06–2.69)
5–9 years old	1.00	1.00*(0.67–1.52)	0.71*(0.45–1.11)	0.92*(0.60–1.40)	1.04*(0.69–1.57)	1.48*(1.01–2.17)	1.45*(0.98–2.14)

*p >0.05, (95% confidence interval)

## Discussion

This is the first study investigating the association of influenza vaccination and childhood otitis media using a population-based analysis. In the current study, using the Taiwan NHIRD, we estimated the secular trends of childhood otitis media before and after the introduction of TIV. The introduction of TIV into the Taiwan childhood immunization program was associated with a significant decline in the incidence of outpatient visits for AOM in the influenza seasons. As is typical of AOM, in the pre-TIV era (2000–2003), children < 2 years had the highest incidence of AOM in the influenza season (98.4 episodes/1000 person-seasons). After the introduction of TIV in 2004–2006, the incidence of outpatient visits with AOM declined significantly (66.1 episodes/1000 person-seasons) in < 2-year-olds, who were enrolled in the free influenza vaccination program launched by the Taiwan CDC. With the increased vaccine coverage rate,^19^ the incidence of outpatient visits for AOM declined correspondingly. In addition of the direct protection provided by the vaccination, we believe that TIV may have induced some herd immunity that further contributed to the reduction in influenza attack rates and the rates of associated AOM in that age group. Compared to the older children who had not received the influenza vaccination, the seasonal AOM incidence increased significantly. Better parental awareness of clinical signs of AOM, increased healthcare quality and accessibility, and better accuracy in the diagnosis of AOM are all considered responsible for this phenomenon.

In the current study, there was no significant change in the incidence of tympanostomy tube insertion in each influenza season between October 2000 and March 2007. Considering the significant reduction in tympanostomy tube insertion after the introduction of the PCV reported in a previous study [20], we consider that the influenza vaccine only protected the immunized children from the preceding viral infection of the middle ear mucosa, but not from the secondary bacterial infection, which was responsible for the recurrent AOM and persistent OME that required tympanostomy tube insertion [21–23].

The pathogenesis of AOM has been studied thoroughly over the previous decades. AOM is strongly seasonal. The incidence of AOM is highest during the fall and winter months and lowest during the spring and summer months, which parallels the incidence of viral URI [24]. Respiratory viruses, such as the influenza virus, respiratory syncytial virus, adenovirus, parainfluenza virus, and rhinovirus, have been isolated from middle ear effusions [13–15]. AOM is commonly caused by bacterial pathogens, such as *Streptococcus pneumoniae*, *Haemophilus influenzae*, and *Moraxella catarrhalis*, either directly or as a complication of a viral URI [25]. Influenza and pneumococcal vaccines are two important commercially available vaccines. In the past three decades, the concept of “prevention is better than cure” has become increasingly popular in the battle between infectious organisms and the human immune system. The efficacy of PCV in preventing AOM has been observed in several studies [20, 26], but the efficacy of influenza vaccines in preventing AOM or tympanostomy tube insertion has not been well studied. In Taiwan, PCV-7 has been available primarily at the recipients’ expense since October 2005 and the cumulative coverage rates for PCV-7 among children < 5 years old in 2005 and 2006 were only 0.7% and 8.6%, respectively [18]. Thus, in the present study, we could evaluate the efficacy of TIV in preventing AOM with limited confounding due to PCVs.

Several studies have addressed the efficacy of TIV in preventing AOM. TIV was found to have a positive effect on AOM by Heikkinen et al. and Clements et al [27, 28]. Both these studies were conducted in day-care centers, with only 187 and 186 children enrolled, respectively. However, Hoberman et al. conducted a randomized double-blind, placebo-controlled 2-year clinical trial in 786 children, aged 6–24 months, including 411 children in the first year and 375 in the second year [29]. The investigators did not identify any significant reduction in the burden of AOM after administration of the TIV. The inconsistent results of these studies may be due to the relatively small sample size and short-term follow-up. In the current study, data from 803,592 children over the period October 2000 through March 2007 were analyzed, and outpatient visits in seven consecutive influenza seasons were studied. The secular trends in AOM incidence were followed before and after the introduction of TIV. This study provided additional evidence of the efficacy of TIV against AOM. The Taiwan CDC has gradually expanded the free influenza vaccination program in recent years. The long-term trends in all clinical manifestations of influenza disease and related complications should be monitored continuously.

An important limitation of our study is that we do not have microbial culture results that could confirm influenza infection for AOM cases. Aspiration of the middle ear fluid in children with AOM, either for microbiology cultures or symptomatic relief, is not part of routine medical care in Taiwan. In addition, laboratory data and culture results are not available in the NHIRD. Second, the patients’ vaccination histories are not comprehensively recorded in the NHIRD. In Taiwan, the free influenza vaccination is not included in the regular childhood immunization schedule, and parents may decide not to have the influenza vaccination administered if they have any concerns. Thus, we were unable to ascertain the immunization status in our study subjects. Third, several risk factors predisposing to AOM have been reported; these include age, socioeconomic status, tobacco smoke exposure, lack of breast-feeding, siblings, and day-care attendance [30]. However, most of this information is not disclosed in the NHIRD; therefore, we could not investigate whether some children have risk factors predisposing to AOM. Finally, as with all studies analyzing large administrative databases, there is potential for misclassification bias when using diagnostic codes to define AOM. However, such biases are unlikely to affect trends over time and the reduction of AOM cases in the < 2-years-old population in influenza seasons since 2004 can most likely be attributed to TIV introduction.

## Conclusion

A significant reduction in primary care consultations for children < 2 years old was observed after the introduction of TIV in Taiwan in 2004. With the increased vaccine coverage, there was an additional decline in the incidence of AOM in 2005 and 2006. These reductions were observed only in vaccine-eligible children, but not in older children, who were not enrolled in the influenza vaccination program.
